# Higher Education, Happiness, and Residents' Health

**DOI:** 10.3389/fpsyg.2020.01669

**Published:** 2020-07-21

**Authors:** Hong Tan, Jin Luo, Ming Zhang

**Affiliations:** ^1^School of Economics and Management, Chongqing Youth Vocational and Technical College, Chongqing, China; ^2^School of Political Science and Public Administration, Southwest University, Chongqing, China

**Keywords:** higher education, happiness, health, semiparameter estimation, shapley value decomposition

## Abstract

The study proposes a new mechanism by which higher education affects the health of residents, showing that higher education can first improve the happiness of residents and then improve their health. In this research, we employ the data collected in Chinese General Social Survey in 2013 and adopt the semiparametric estimation methodology of ordered probit model. Our main findings include the following. First, compared with the residents without higher education, residents with high education enjoy better health conditions, and residents' happiness also significantly affects their health conditions. Second, higher education may have a long-term impact on residents' health by affecting their happiness. Third, the results of grouping test demonstrate that, with the increase in age, the influence of residents' happiness on health is more pronounced, but the mechanism of higher education to improve health status by improving residents' happiness becomes unobvious. Furthermore, we adopt the Shapley value decomposition methodology to decompose the effects of various factors on residents' health. We find that with the increase in age, happiness contributes more and more to residents' health conditions.

## Introduction

As a capital of human resources, education is not only an important indicator of social and economic status but also has a direct impact on one's occupation, income, and wealth. Therefore, among the various social factors that affect health and cause health inequality, it is sometimes regarded as the root cause of health disparity. Numerous studies have revealed a robust positive correlation between education and health. The existing literature shows that education mainly affects residents' health through the following three means. First, the more educated an individual is, the higher his or her income level will be (Moen, 1999). Because the living standard of the higher-income group is also higher, it is often accompanied by better health (Bai et al., 2020). Second, people with higher education level are more likely to make better use of health care and medical information, adapt to complex medical treatment, and thus benefit more from the improvement of medical technology (Glied and Lleras-Muney, 2003). Third, better education will help people better understand the potential harms caused by bad life behaviors on health, encourage people to develop healthy living habits such as minimize or quit smoking, and develop good eating and exercise habits (Kemptner et al., 2011).

Since the twenty-first century, with improving quality of health care and level of people's education, the health status of residents in developing countries has been significantly improved. However, compared with developed countries, there is still a certain gap. As the most populous developing country, with the introduction of Healthy China Strategy, the issue of Chinese national health has garnered extensive attention. According to Chinese Family Health Big Data Report 2018, in recent years, Chinese people's awareness of healthy life and family health management has been enhanced, and the health concept of active prevention has been embraced by people. However, more and more young people have health problems, and the rapid growth of the elderly unhealthy people has become national health issues. Many scholars discuss how to improve the health level of Chinese people from the perspective of education. Zhao and Hou (2005), Zhao (2006), Li and Feng (2006), and Cheng et al. (2015) all showed that the improvement of education level can increase income and improve residents' living behaviors, thus significantly and positively impacting the health of Chinese citizens. Considering selective bias and heterogeneity, Wang and He (2015) estimated the impact of high school education, and higher education on health using Propensity Score Matching. Their results obtained by local linear matching confirmed that higher education can produce better health level. Zheng and Zeng (2018) used tracking data to study the health return of education and found that the health return of education gradually increased in recent years. Li and Liu (2019) tested the causal relationship between Chinese education and national health and health behaviors based on the two-stage least squares method (2SLS). They found that the improvement of education level significantly improved the men's health condition. However, some scholars question the positive health effects of higher education in China. Zhao and Hu (2016) believed that, as a developing country, China's higher education fell behind developed countries in terms of curriculum, sports facilities, and health concept, and residents with higher education tended to engage in more stressful work, which made people have irregular rest and work schedule and suffer from extensive use of electronic radiation products. Thus, higher education would have a significant negative impact on health.

To some extent, these studies revealed the health effects of higher education in China. However, they typically ignore another potential way of higher education affecting the health of residents, which maybe a very important reason for the inconsistency of existing research results. In fact, higher education can also affect the health of residents by affecting their well-being. A number of studies based on Chinese data have found that education can improve residents' well-being. First, education improves the subjective well-being of residents by improving their income level (Luo, 2006). Second, people receiving higher education are better at communication and can deal with disputes and contradictions flexibly (Gilar-Corbí et al., 2018; Zhao et al., 2019). This not only makes for expanding and maintaining social relations with others but also enables individuals to obtain a stable emotional support and strengthen their perception of happiness (Huang, 2013). Finally, education can improve self-identity, so can promote happiness (Zhou, 2015). If it is real that education affects residents' well-being, education is likely to further affect residents' health because there is a lot of evidence that happiness has a positive impact on residents' health based on China and other countries. Graham et al. (2004) took the survey data of Russian residents as a sample to investigate the impact of happiness on residents' health. They found that those residents with higher happiness had stronger optimism attitude and enjoyed better health in the future. Straume and Vitters (2015), Sabatini (2014), and Siu et al. (2015) confirmed that the residents with higher happiness were healthier. They argued that people's optimism could regulate their physical functions. Based on Chinese data, Zhu and Yang (2017) found that individuals with high happiness may easily form good habits for health, such as eating a balanced diet and exercising regularly; thus, happiness had a positive impact on health.

This study believes that education can improve residents' happiness and thus improve their health level. Based on the data of Chinese General Social Survey in 2013, this study empirically investigates the impact of higher education on health condition of residents by using the semiparametric estimation methodology of ordered probit model. It shows that higher education can improve health condition by affecting residents' happiness. Furthermore, in order to investigate the impact of higher education on the health of residents in different age, we will conduct grouping test.

## Data Description

Our research data is from 2013 Chinese General Social Survey (CGSS), a large-scale nationwide survey conducted by the Department of Sociology, Renmin University of China. Because the paper mainly focuses on the impact of higher education on residents' health, we need delete the samples with missing critical information. As a result, 10,037 observations were obtained.

**Figure 1:**
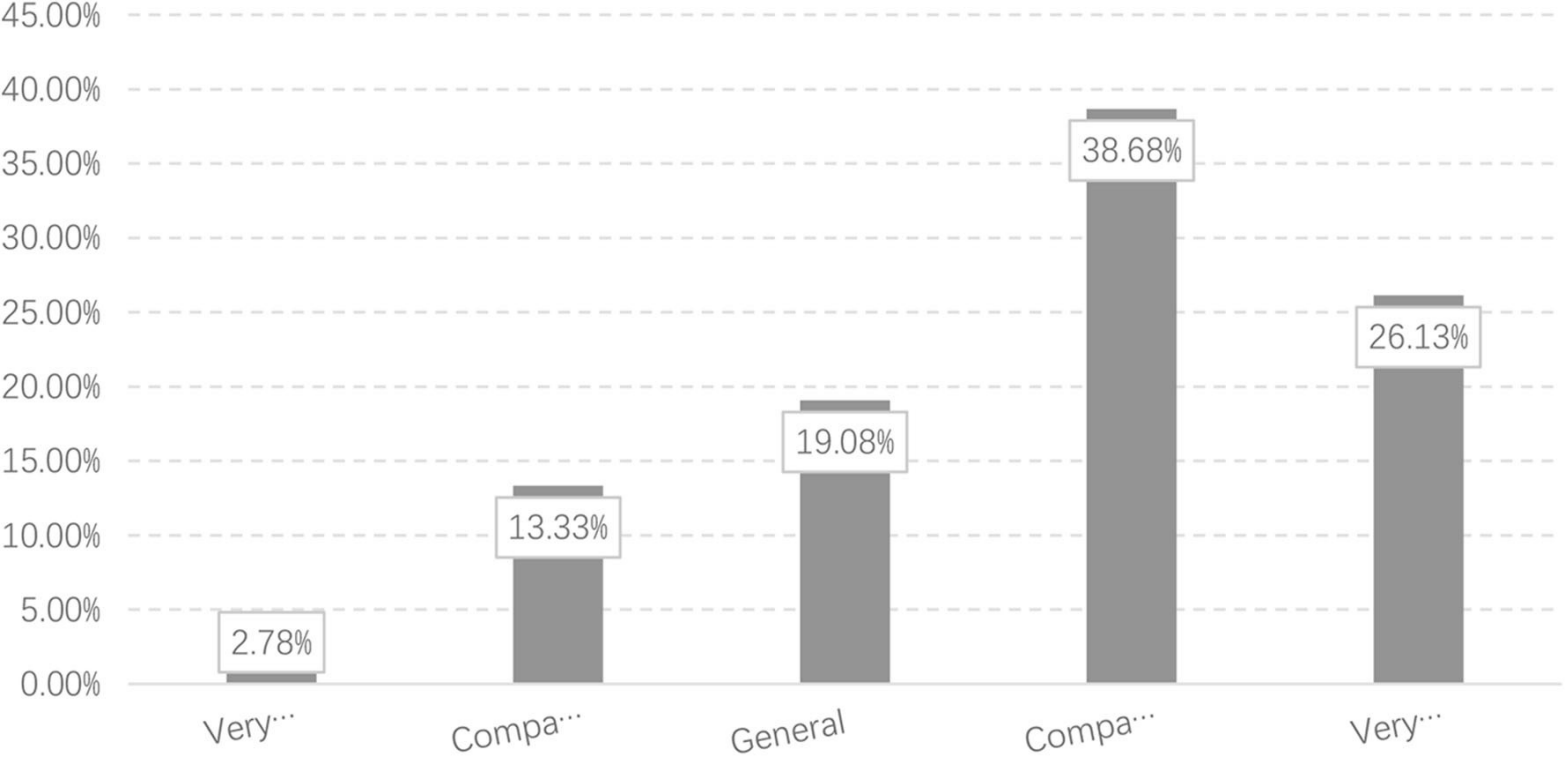
Health conditions of survey respondents.

Residents' health status, labeled as health, is the core dependent variable in this paper, which is a multidimensional concept including physiological health and psychological health. The existing literature often measures it by subjective self-evaluation, which is also adopted in this research. In the CGSS questionnaire, a main question employed in our research is: “what do you think your physical health is?” The answer to the question employs the 5-point Likert scale in which values of 1 and 5 represent “very unhealthy” and “very healthy,” respectively. Figure 1 shows, in the survey, 2.78% of the residents consider themselves very unhealthy, and 13.33% consider themselves comparatively unhealthy; 38.68% of the residents think they are in good health, and 26.13% think they are very healthy. This implies that most respondents' self-perception of physical condition is healthy.

**Figure 2:**
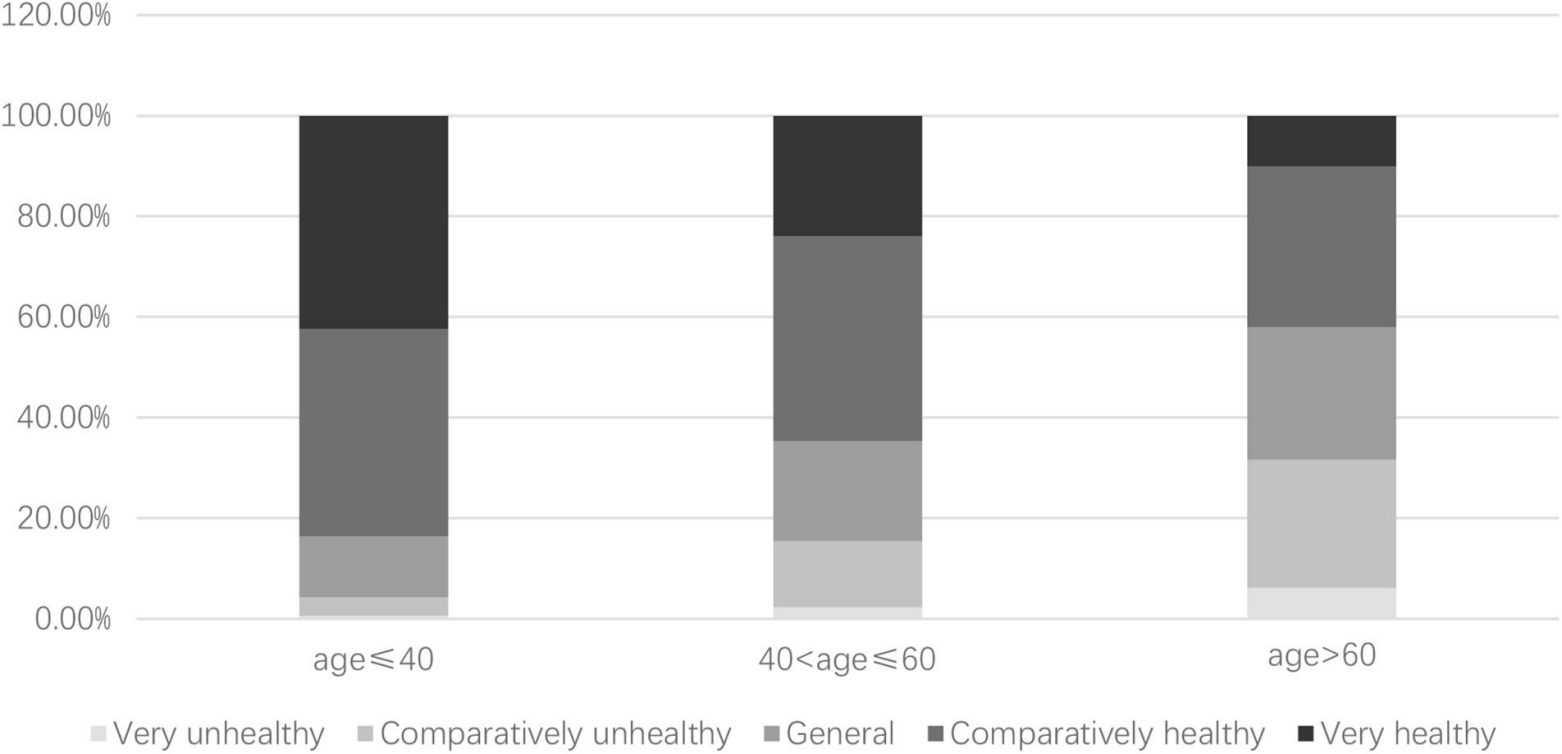
Health conditions among residents of different age groups.

Because age is an important factor that influences health condition, Figure 2 further reports the differences in health condition among residents of different age groups. We divide our survey subjects into three groups according to their age: age ≤ 40, 40 < age ≤ 60, and age > 60. In our samples, The proportions of the three groups are 33.12, 41.42, and 25.47%, respectively. As shown in Figure 2, with the increase in age, the health condition of residents tends to deteriorate. About 80% of residents under the age of 40 think they are in good health, while only about 50% of residents over the age of 60 think they are in good health.

The study focuses on the impact of higher education on residents' health status, so it is necessary to check whether a resident received higher education. We use the core explanatory variable *education* in this study. We obtain the educational attainment of the respondents based on the answer to the question, “your highest education level at present” in the CGSS questionnaire. According to the respondents' answers, when a respondent chooses “junior college (adult higher education),” “undergraduate college (regular higher education),” “undergraduate degree (adult higher education),” and “graduate and above,” s/he is considered to have received higher education. We assign it with a value of 1. In contrast, when a respondent chooses “no education,” “private school,” “primary school,” “junior high school,” “vocational high school,” “ordinary high school,” “technical secondary school,” or “technical school,” s/he is considered to have not received higher education. We assign it with a value of 0. Among our respondents, 1,595 respondents are receiving or have received higher education, accounting for 15.89%. The remaining 8,742 respondents are non-higher education receivers, accounting for 84.11%.

In this study, we argue that higher education can improve residents' health by improving their sense of happiness. Next, we need to know the happiness assessment of different residents. We measure the variable happiness from the survey question: “in general, how do you feel about your own life?” The answer to the question is also designed to utilize 5-point Likert scale in which values 1 and 5 represent very unhappy and very happy, respectively. Of the respondents, 59.18% were happy and 13.79% were very happy.

In order to accurately judge the impact of higher education on residents' health status, we designed an econometric model for empirical testing. In addition to higher education, income level, sex, age, marriage status, job industry, and other factors may also affect individuals' perception of happiness. Hence, we also need to control these variables based on the CGSS questionnaire. Based on the answers to the question, “what was your total income last year (2012)?” we obtain the values of the variable *income*. The value of the variable *sex* is 1 for male and 0 for female. The variable *age* is assigned as the respondent's age in 2013. As for the variable *marriage*, residents with “cohabitation” and “married' status are denoted as 1, while the others are denoted as 0. Based on the answers to the question, “what is your work experience and status?” we obtain the values of the variable *work*. For respondents in the non-agricultural industry, we assign the value of 1. Otherwise, we assign the value of 0. As shown in Table 1, we report main variables' descriptive statistics, such as mean, standard deviation, minimum, and maximum.

**Table 1:** Descriptive statistics of the main variables.

**Variable**	**Mean**	**SD**	**Min**	**Max**
Health	3.7205	1.0759	1	5
Education	0.1589	0.3656	0	1
Happiness	3.7363	0.9378	1	5
Age	48.9565	16.0763	17	97
Income	2.3873	3.6952	0	100
Sex	0.5108	0.4999	0	1
Marriage	0.8065	0.3950	0	1
Work	0.6981	0.4591	0	1

## Estimation Method and Results

### Semiparameter Estimation Method of Ordered Probit Model

According to the questionnaire of CGSS 2013, we can adopt the maximum likelihood method of the ordered probit model because health condition is an ordered dependent variable. However, due to the fact that health conditions are subjective indicators, it is often difficult for an individual to judge them accurately. If those latent variables are grouped precisely according to the integers from 1 to 5, there is reliability deviation. Therefore, the explained variables are redivided as:

(1) $$\begin{array}{l} s^{*}=\{\begin{array}{cc} 1 & s<3\\ 2 & s=3\\ 3 & s>3 \end{array} \end{array}$$

By transforming the above equation, *s* can be redivided into three non-overlapping intervals, s^*^. The new variable *s*′ is further normalized as follows:

(2) $$\begin{array}{l} s^{*}=\{\begin{array}{cc} 1 & s^{\prime }<\lambda_{1}\\ 2 & \lambda_{1}\leq s^{\prime }\lambda_{2}\\ 3 & s^{\prime }\geq \lambda_{2} \end{array} \end{array}$$

For Equation (2), the probability of s^*^ taking a particular value can be calculated as follows:

(3) $$\begin{array}{l} pr\left(s^{*}=j\right)=\{\begin{array}{ll} \;F(\lambda_{1}-x_{i}{\;}^{\prime }\beta )\; & j=1\\ F(\lambda_{2}-x_{i}{\;}^{\prime }\beta )-F(\lambda_{1}-x_{i}{\;}^{\prime }\beta ) & \;j=2\\ \;1-F(\lambda_{2}-x_{i}{\;}^{\prime }\beta ) & j=3\; \end{array}\; \end{array}$$

In Equation (3), *F*(•) is subject to normal distribution, λ_1_and λ_2_ are the new interval partition value, *x* represents explanatory variables including higher education, and β represents the corresponding estimated coefficient. Next, s^*^ is taken as the explained variable to establish the ordered probit model. Then, the logarithmic likelihood function of this model is:

(4) $$\begin{array}{l} \ln\;L\left(\beta ,\lambda_{1},\lambda_{2},\lambda_{3}\right)=\overset{n}{\underset{i=1}{\sum }}\overset{3}{\underset{j=1}{\sum }}1\left\{s^{*}=j\right\}\ln\;[F\left(\lambda_{j+1}-x_{i}^{{\;}^{\prime }}\beta \right)\\ \;-F(\lambda_{j}-x_{i}^{{\;}^{\prime }}\beta )] \end{array}$$

In Equation (4), 1{•} denotes the indicative function: it equals 1 when the condition in parentheses is satisfied; otherwise, it is 0. The coefficientsβ and the parameters λ_1_ and λ_2_ of the ordered response model can be estimated by maximizing the log-likelihood function. The commonly used ordered probit model assumes that the residuals follow a normal distribution, which is, however, often difficult to check. Stewart (2004) proposed that the semiparametric method could be used for correction. Assuming that the distribution function of ε is unknown, the ε density function can be simulated by the Hermit sequence $f_{k}(\varepsilon )=1/\alpha *({\sum_{\rho =0}^{k}\gamma_{s}\varepsilon^{2})}^{2}\prod \left(\varepsilon \right)$. Furthermore, we can obtain the values of other parameters. It can be verified that when *k* ≥ 2, the estimated coefficient is the same as that of the ordered probit model (Stewart, 2004). Therefore, *k* = 3 is the starting point of semiparameter estimation. In addition, given that the nested nature of semiparameter estimation, the likelihood ratio test (LR test) can be used to determine the necessity of semiparameter estimation.

### Analysis of Estimated Results

To test the impact of higher education on residents' health, we established an econometric model, and the estimated results are shown in Table 2. The models (1) and (2) in Table 2 are the estimated results of the traditional ordered probit model. In model (1), we only controlled the *age* variable apart from introducing the core explained variable *education*. Based on model (1), model (2) introduces variables such as *happiness, income, sex, marriage*, and *work*. The estimated coefficients of higher education variables in models (1) and (2) are significantly positive, indicating that residents with higher education have better health conditions. Since the general ordered probit model may have reliability deviation, models (3) and (4) are the estimation results of the semiparameter estimation method of the ordered probit model. LR test rejects the null hypothesis of ordinary parametric model, indicating that the semiparametric model is better. Such model needs to select the appropriate value of the parameter *k* in the residual distribution function. To this end, we increase *k* continuously from 3. Because the model corresponding to low-order *k* is nested in the model corresponding to high order *k*, LR test can be used to determine the appropriate *k* value. The nested LR test of both models shows that there is no significant difference in the estimated results of the model between *k* of 4 and 5, but there is significant difference in the estimated results of the model between *k* of 3 and 4, so the final value of *k* should be 4. Models (3) and (4) are the estimation results when *k* is 4. The estimated coefficient of *education* variable in the models (3) and (4) is still significantly positive, indicating that residents with higher education do have better health condition compared with those without higher education, which is basically consistent with our expectation. In Table 2, the estimated coefficient of *happiness* variable is also significantly positive, indicating that residents' *happiness* will also affect their subjective health assessment. The estimated coefficient of *age* variable is negative and significant at the level of 1%, indicating that the health condition of residents will deteriorate with age. The estimated coefficients of *income, sex, marriage*, and *work* are also significantly positive, which is also in line with our expectations.

**Table 2:** A test of high education's influence on residents' health.

	**(1)**	**(2)**	**(3)**	**(4)**
Education	0.2724 (6.76)^***^	0.1106 (2.56)^**^	0.3224 (4.58)^***^	0.1144 (2.73)^***^
Age	−0.0387 (−7.89)^***^	−0.0496 (−9.44)^***^	−0.0474 (−4.15)^***^	−0.0394 (−3.76)^***^
Happiness		0.2782 (6.38)^***^		0.2697 (3.84)^***^
Income		0.0313 (6.17)^***^		0.0237 (3.12)^***^
Sex		0.0822 (3.16)^***^		0.0750 (2.74)^***^
Marriage		0.1720 (4.87)^***^		0.1608 (3.56)^***^
Work		0.2568 (8.77)^***^		0.2536 (4.63)^***^
Log likelihood	−8256.7562	−8133.5294	−8255.1981	−8130.6683
*P*-value (LR)			0.0605	0.0167
Standard deviation			1.1201	1.0637
Skewness			−0.1016	0.6425
Kurtosis			2.6976	3.7317

*, **, and *** *are significant at the 10, 5, and 1% levels, respectively. T-statistics is reported in brackets. The table reports LR test of ordinary OP model only, and P-value results from LR test*.

Next, the paper needs to test whether higher education can improve health condition by affecting residents' happiness. Following Mackinnon et al. (1995) and Preacher and Hayes (2008), we compare the coefficient of variable *education* in the model including variable *happiness* with that in the model excluding variable *happiness*, and it is widely assumed that *education* can exert effect on *health* by *happiness* when the coefficient in the model excluding variable *happiness* is bigger than that in the model including variable *happiness*. The models (5) and (6) in Table 3 are the estimation results by using the general ordered probit model, and the results show that the coefficient of variable *education* become bigger when we drop variable *happiness*. Models (7) and (8) report the results by using semiparameter estimation method of ordered probit model. LR test shows that using the semiparametric estimation method is better. Nested LR test indicates that the model residual sequence *k* is 4. We still find that the coefficient of variable *education* becomes bigger when we drop variable *happiness*. Consequently, we can make sure *education* can exert effect on *health* by *happiness*.

**Table 3:** The mechanism test based on the new method.

	**(5)**	**(6)**	**(7)**	**(8)**
Education	0.1106 (2.56)^**^	0.1414 (3.32)^***^	0.1144 (2.73)^***^	0.1422 (2.17)^**^
Happiness	0.2782 (6.38)^***^		0.2697 (3.84)^***^	
Age	−0.0496 (−9.44)^***^	−0.0256 (−29.94)	−0.0394 (−3.76)^***^	−0.0258 (−3.49)^***^
Income	0.0313 (6.17)^***^	0.0300 (5.96)^***^	0.0237 (3.12)^***^	0.0272 (4.36)^***^
Sex	0.0822 (3.16)^***^	0.0832 (3.20)^***^	0.0750 (2.74)^***^	0.0878 (2.39)^**^
Marriage	0.1720 (4.87)^***^	0.1038 (3.19)^***^	0.1608 (3.56)^***^	0.1254 (2.56)^**^
Work	0.2568 (8.77)^***^	0.2350 (8.07)^***^	0.2536 (4.63)^***^	0.2524 (3.12)^***^
Log likelihood	−8133.5294	−8138.1288	−8130.6683	−8127.4898
*P* value (LR)			0.0167	0.0000
Standard deviation			1.0637	1.5878
Skewness			0.6425	0.6411
Kurtosis			3.7317	2.3779

*, **, and *** *are significant at the 10, 5, and 1% levels, respectively. T-statistics is reported in brackets. The table reports LR test of ordinary OP model only, and P-value results from LR test*.

## Grouping Test and Contribution Decomposition

### Grouping Test

As age is an important factor affecting the health of residents, the health status characteristics of residents in different ages are quite different. Therefore, we will divide our survey subjects into three groups according to their age: age ≤ 40, 40 < age ≤ 60, and age > 60. The proportions of respondents who are receiving or have received higher education are 31.89, 9.29, and 5.75% respectively. Table 4 reports the results obtained by using the semiparametric estimation method of ordered probit model. LR test shows that using the semiparametric estimation method is better. Nested LR test indicates that the model residual sequence *k* is 4. Models (9) and (10) in Table 4 is the impact effect's estimation of higher education. The estimated coefficient of the *education* variable was significantly positive in the group age ≤ 40, suggesting that residents with higher education had better subjective health assessment for this group. The estimated coefficient of *education* variable was positive but not significant in the group 40 < age ≤ 60 but negative in the group age > 60. Furthermore, if we observe estimates coefficient of *happiness* variable, we will find that the estimated coefficient changing trend with age appear contrary to *education* variable. The estimated coefficient of this variable was significantly positive in the group age ≤ 40. With the increase in age, the estimated coefficient of this variable not only tends to become significant but also become large. This result indicates that, with the growth of age, the people who received higher education may suffer from irregular rest schedule (Zhao and Hu, 2016). Hence, the influence of higher education on residents' health status will be increasingly weakened, while the influence of psychological factors such as happiness perception on residents' self-rated health status will be more pronounced.

**Table 4:** Grouping test results I.

	**(9)** **Age ≤ 40**	**(10)** **40 < age ≤ 60**	**(11)** **Age > 60**
Education	0.1608 (1.84)^*^	0.0190 (0.26)	−0.0138 (−0.53)
Happiness	0.2265 (1.75)^*^	0.2680 (5.37)^***^	0.3086 (3.21)^***^
Age	−0.0901 (−1.46)	−0.0163 (−0.25)	−0.1451 (−2.43)^**^
Income	0.0066 (1.06)	0.0557 (6.58)^***^	0.0854 (4.60)^***^
Sex	0.0966 (1.78)^*^	0.0962 (2.43)^**^	0.0739 (1.56)
Marriage	0.1949 (2.57)^**^	0.3022 (4.55)^***^	0.0680 (1.26)
Work	0.2748 (3.91)^***^	0.1878 (4.27)^***^	0.1864 (3.38)^***^
Log likelihood	−1721.3885	−3622.9714	−2700.8365
*P* value (LR)	0.0051	0.0000	0.0000
Standard deviation	1.9525	1.8988	1.5421
Skewness	−0.3272	0.4643	0.4422
Kurtosis	1.7571	2.1186	3.1578

*, **, and *** *are significant at the 10, 5, and 1% levels, respectively. T-statistics is reported in brackets. The table reports LR test of ordinary OP model only, and P-value results from LR test*.

As shown in Table 5, we further compare the coefficient of variable *education* in models (12)–(14) with that in models (9)–(11) excluding variable *happiness*. Table 5 shows that, in the group age ≤ 40, coefficient of variable *education* become bigger when we drop variable *happiness*. However, in the group 40 < age ≤ 60 or age > 60, coefficient of variable *education* change little when we drop variable *happiness*. It means that, with the growth of age, mechanism of higher education to health status by improving residents' happiness become less salient. This is similar to the results in Table 4. With the growth of age, the people who received higher education may suffer from irregular rest schedule (Zhao and Hu, 2016). As a result, this reduces people's positive feeling.

**Table 5:** Grouping test results II.

	**(12)** **Age ≤ 40**	**(13)** **40 < age ≤ 60**	**(14)** **Age > 60**
Education	0.2558 (2.28)^**^	0.0183 (0.24)	−0.1610 (−0.83)
Age	−0.0387 (−4.96)^***^	−0.0218 (−3.34)^***^	−0.0249 (−4.90)^***^
Income	0.0327 (3.35)^***^	0.0785 (5.65)^***^	0.2132 (3.03)^***^
Sex	−0.1255 (−1.63)	0.0892 (1.97)^**^	0.0870 (1.00)
Marriage	0.1083 (1.07)	0.2880 (3.50)^***^	0.0744 (0.79)
Work	0.3094 (3.35)^***^	0.2242 (2.90)^***^	0.2786 (2.84)^***^
Log likelihood	−1788.0027	−3617.5107	−2690.1974
*P*-value (LR)	0.0002	0.0001	0.0000
Standard deviation	1.9428	2.0010	1.8666
Skewness	−1.5080	0.0964	−0.3501
Kurtosis	4.6272	1.8673	2.3516

*, **, and *** *are significant at the 10, 5, and 1% levels, respectively. T-statistics is reported in brackets. The table reports LR test of ordinary OP model only, and P-value results from LR test*.

In order to make sure grouping test results are robust, we retest the estimation by dropping variable *age*. On this condition, the results in models (15)–(20) in Table 6 show that, with the growth of age, the change in coefficients of variable *education* before and after dropping happiness is small. This means that the mechanism of higher education to health status by improving residents' happiness becomes less salient in elderly group.

**Table 6:** Grouping test results III.

	**(15)** **Age ≤ 40**	**(16)** **40 < age ≤ 60**	**(17)** **Age > 60**	**(18)** **Age ≤ 40**	**(19)** **40 < age ≤ 60**	**(20)** **Age > 60**
Education	0.2107 (1.85)^*^	0.0517 (0.72)	−0.1453 (−0.81)	0.2481 (2.42)^**^	0.0578 (0.78)	−0.0179 (−0.07)
Happiness	0.3023 (2.69)^***^	0.3529 (6.90)^***^	0.4572 (5.50)^***^			
Income	0.0024 (2.24)^**^	0.0632 (4.76)^***^	0.1238 (3.82)^***^	0.0255 (2.29)^**^	0.0796 (6.99)^***^	0.2718 (7.53)^***^
Sex	0.1311 (1.77)^*^	0.0921 (2.34)^**^	0.2071 (2.64)^***^	−0.1578 (−1.75)^*^	0.0724 (1.79)^*^	0.0252 (0.31)
Marriage	−0.1934 (−1.42)	0.1752 (2.71)^***^	0.1481 (1.47)	−0.2293 (−2.40)^**^	0.2873 (4.55)^***^	0.1466 (1.74)^*^
Work	0.2821 (1.67)^***^	0.1792 (3.65)^***^	0.2937 (2.89)^***^	0.3698 (2.48)^**^	0.2418 (4.33)^***^	0.2451 (2.38)^**^
Log likelihood	−1925.4324	−3524.9133	−2563.1475	−1805.8096	−3634.4103	−2693.4222
*P*-value (LR)	0.0001	0.0000	0.0000	0.0001	0.0000	0.0000
Standard deviation	1.1736	1.8933	2.0413	1.5089	2.0989	2.3351
Skewness	−0.3732	0.4331	0.5807	−1.4094	−0.0558	−0.3307
Kurtosis	3.0074	1.9891	2.5003	4.3405	1.6487	1.8647

*, **, and *** *are significant at the 10, 5, and 1% levels, respectively. T-statistics is reported in brackets. The table reports LR test of ordinary OP model only, and P-value results from LR test*.

### Contribution Decomposition

The coefficient estimation results show that higher education has a complex impact on residents' health status. Furthermore, we will evaluate the importance of *education* variables by decomposing contribution rate. Since the multiple explanatory variables considered in this study include *education, happiness*, and *age*, there may be multicollinearity among these variables. This may lead to unreliable results using traditional decomposition methods based on regression coefficients (Fields, 2003). The newly developed Shapley value decomposition method can effectively overcome this problem, as it can measure the marginal contribution and contribution rate of explanatory variables (Israeli, 2007; Huettner and Sunder, 2012). The basic idea is that, when measuring the contribution of an explanatory variable, we first calculate the *R*^2^ in the model that contains the explanatory variable and then remove the variable to observe the change in *R*^2^. The larger the change in *R*^2^ is, the higher contribution rate of the variable is. Because there may be many different combinations of explanatory variables, Israeli (2007) and Huettner and Sunder (2012) suggest taking the average value of various combinations to obtain the marginal contribution or contribution rate.

For this study, the following is the main idea of Sharpley value decomposition. In the econometric model in this study, there are seven variables affecting health condition, which are denoted as *x*. Hence, *x* has 7! possible combinations, denoted as set Θ. Let us assume θ ∈ Θ is one from the set. When measuring contribution rate of *edu* variable, we assume that θ_*edu*_ is the position of *education* variable in the set θ, *P*(θ, θ_*edu*_) is the set of variable set who is placed in front of θ_*edu*_, denoted as *P*(θ, θ_*edu*_) = {*x* ∈ *X*|θ_*x*_ < θ_*edu*_}. Then, marginal contribution of *education* variable can be obtained by the following equation:

(5) $$\begin{array}{l} mc_{edu}=R^{2}\left[P\left(\theta ,\theta_{edu}\right)\cup edu\right]-R^{2}\left[P\left(\theta ,\theta_{edu}\right)\right] \end{array}$$

In Equation (5), *R*^2^(•) represents *R*^2^ of the regression equation, and *mc*_*edu*_ represents the marginal contribution of *education* variable. Furthermore, the contribution rate of *education* variable can be defined as follows:

(6) $$\begin{array}{l} s_{edu}=mc_{edu}/R^{2}\left[P\left(\theta ,\theta_{edu}\right)\cup edu\right] \end{array}$$

In the equation above, *S*_*edu*_ is the contribution rate of *education* variable. Then, we need to calculate *S*_*edu*_ under various combinations so as to obtain the average contribution rate.

(7) $$\begin{array}{l} S_{edu}=\overset{\;}{\underset{\theta \in \Theta }{\sum }}s_{edu}/7! \end{array}$$

Using the Sharpley value decomposition method, we obtained the contribution rate of *education* variable and other variables affecting residents' health status. The results are shown in Table 7. In the overall sample, *age* variable is the most important factor affecting physical condition, and contribution rate is 60.12%. The second is residents' happiness, with a contribution rate of 14.00%, indicating that residents' subjective happiness is also an important factor affecting their health condition. The contribution rate of higher education (*education*) is 5.56%, ranking the fifth in the seven variables, indicating that the impact of higher education is relatively small. Table 7 shows that main influencing factors vary with the growth of age. The contribution rate of *education* and *happiness* variables are, respectively, 10.92 and 38.49% in the group age ≤ 40, and 4.42 and 39.62% in the group 40 < age ≤ 60. For residents who are older than 60, the contribution rate of *education* variable was 1.69%, and the contribution rate of *happiness* variable was 45.12%. Similar to the estimated results using the semiparametric estimation method of ordered probit model, the Shapley value decomposition results show that with the growth of age, the impact of higher education on residents' health tends to be weakened, and the impact of residents' subjective happiness and other psychological factors on physical health becomes more and more important.

**Table 7:** Decomposition of contribution rate (%).

	**Whole sample**	**Age ≤ 40**	**40 < age ≤ 60**	**Age > 60**
Education	5.56	10.92	4.42	1.69
Age	60.12	29.03	17.92	8.59
Happiness	14.00	38.49	39.62	45.12
Income	5.70	4.32	12.91	21.24
Sex	1.03	1.85	4.47	3.92
Marriage	0.99	3.29	5.43	2.51
Work	12.61	12.10	15.23	16.93

## Conclusions and Policy Recommendations

Based on the sample data obtained from Chinese General Social Survey (CGSS) in 2013, our study empirically investigates the impact of higher education on health condition of residents. For empirical testing, we adopt the semiparametric estimation methodology of ordered probit model. Our empirical results show that residents with higher education are indeed in better health conditions than those without higher education. Furthermore, residents' happiness significantly affects their health conditions. Moreover, because higher education will affect residents' happiness perception, it will have a long-term impact on residents' health status. We conduct grouping tests, and the results show that with the increase in age, the influence of residents' happiness on subjective health assessment is more salient. However, the mechanism by which higher education improves health status by improving residents' happiness become less salient. Finally, our results of Sharpley value decomposition also demonstrate that happiness is increasingly more important to residents' health with the increase in age.

In short, our empirical results reveal that higher education has a significant and positive causal effect on health conditions. Our results lead to the following policy insights. First, improving education level is an important way for developing countries to improve their citizens' health conditions. Therefore, in developing countries, it is necessary to continue to develop higher education, especially to provide fair education opportunities for vulnerable groups. Second, with the growth of age, the mechanism of higher education to health status by improving residents' happiness become less significant. This means that it is useful to help younger people learn more about health management in order to minimize their future unhealthy lifestyles. Third, our results confirm that happiness is a critical factor that influences health in elderly group. Since happiness is a subjective feeling to a large extent, it is not only necessary for a developing country to provide various economic support policies for elderly citizens but also to develop a national psychological counseling service system.

## Data Availability Statement

Publicly available datasets were analyzed in this study. This data can be found here: http://cgss.ruc.edu.cn/.

## Author Contributions

HT conceived the main idea of the theoretical mechanism and manuscript drafting. JL did the literature review and related empirical analysis. MZ provided main idea of the revised manuscript. All authors contributed to the article and approved the submitted version.

## Conflict of Interest

The authors declare that the research was conducted in the absence of any commercial or financial relationships that could be construed as a potential conflict of interest.
